# Testing the AC/DC hypothesis: Rock and roll is noise pollution and weakens a trophic cascade

**DOI:** 10.1002/ece3.4273

**Published:** 2018-07-10

**Authors:** Brandon T. Barton, Mariah E. Hodge, Cori J. Speights, Anna M. Autrey, Marcus A. Lashley, Vincent P. Klink

**Affiliations:** ^1^ Department of Biological Sciences Mississippi State University Mississippi State Mississippi; ^2^ Department of Wildlife, Fisheries, and Aquaculture Mississippi State University Mississippi State Mississippi

**Keywords:** aphids, biological control, global change, lady beetles, predator‐prey interaction, sound pollution, soybean, trophic cascade

## Abstract

Anthropogenic sound is increasingly considered a major environmental issue, but its effects are relatively unstudied. Organisms may be directly affected by anthropogenic sound in many ways, including interference with their ability to detect mates, predators, or food, and disturbances that directly affect one organism may in turn have indirect effects on others. Thus, to fully appreciate the net effect of anthropogenic sound, it may be important to consider both direct and indirect effects. We report here on a series of experiments to test the hypothesis that anthropogenic sound can generate cascading indirect effects within a community. We used a study system of lady beetles, soybean aphids, and soybean plants, which are a useful model for studying the direct and indirect effects of global change on food webs. For sound treatments, we used several types of music, as well as a mix of urban sounds (e.g., sirens, vehicles, and construction equipment), each at volumes comparable to a busy city street or farm tractor. In 18‐hr feeding trials, rock music and urban sounds caused lady beetles to consume fewer aphids, but other types of music had no effect even at the same volume. We then tested the effect of rock music on the strength of trophic cascades in a 2‐week experiment in plant growth chambers. When exposed to music by AC/DC, who articulated the null hypothesis that “rock and roll ain't noise pollution” in a song of the same name, lady beetles were less effective predators, resulting in higher aphid density and reduced final plant biomass relative to control (no music) treatments. While it is unclear what characteristics of sound generate these effects, our results reject the AC/DC hypothesis and demonstrate that altered interspecific interactions can transmit the indirect effects of anthropogenic noise through a community.

Rock and roll ain't noise pollution, rock and roll will never die

AC/DC, 1980

## 1 INTRODUCTION

As human populations increase, so do anthropogenic impacts on organisms and ecosystems (Defries, Foley, & Asner, 2004; Ellis, 2011; Hooper et al., 2005). These effects arise in diverse and unexpected ways: spreading invasive species (Hulme, 2009); warming winters and decreasing snow (Penczykowski, Connolly, & Barton, 2017); rising sea levels (Harley et al., 2006); slowing winds (Barton, 2014; Weimerskirch, Louzao, de Grissac, & Delord, 2012); and the list goes on (Gunderson, Armstrong, & Stillman, 2016; Rosenblatt, Smith‐Ramesh, & Schmitz, 2016; Walther et al., 2002). Ecologists have made considerable progress toward understanding how these factors affect species directly, as well as indirectly by altering interactions among species within an ecosystem (Laws, 2017; Tylianakis, Didham, Bascompte, & Wardle, 2008). However, research efforts and progress have not been uniform across anthropogenic disturbances or the taxa affected. Indeed, there is considerable bias in the literature toward a few factors, including temperature, precipitation, and CO_2_ concentrations, with many other abiotic factors relatively unstudied (Barton, 2017).

Among the least studied aspects of global change are the ecological effects of anthropogenic sound. Anthropogenic sound is increasingly recognized as a major component of global change in both urban and rural environments (Buxton et al., 2017), but its consequences for species and their interactions remain relatively unknown. Sound is an important part of many species life histories, and consequently, anthropogenic sounds can disrupt these activities and processes (Barber, Crooks, & Fristrup, 2010; Francis, Ortega, & Cruz, 2009; McMahon, Rohr, & Bernal, 2017). While some studies have explored the effects of noise at the individual or intraspecific level, considerably less is known about how anthropogenic sound can influence interactions among species in a community (McMahon et al., 2017). Those studies that have evaluated interspecific effects of anthropogenic sound largely focused on vertebrates that use sound to hunt or find mates, such as bats, birds, and frogs (Francis et al., 2009; Luo, Siemers, & Koselj, 2015; Simpson et al., 2016). Almost nothing is known about the effects of sound pollution on some of the most abundant animals, insects.

In a review of terrestrial sound pollution literature, Morley, Jones, and Radford (2014) documented that only two of 83 papers (Lampe, Schmoll, Franzke, & Reinhold, 2012; Shieh, Liang, Chen, Loa, & Liao, 2012) considered invertebrate species, although more insect‐related articles have been published recently (Costello & Symes, 2014; McMahon et al., 2017; Orci, Petróczki, & Barta, 2016; Schmidt, Morrison, & Kunc, 2014). In general, these papers focus on how acoustic communication performed by insects (e.g., courtship behaviors) is impacted by anthropogenic sound (Morley et al., 2014). While there is evidence of direct, intraspecific effects of sound on insects and other taxa, consequent indirect effects that can arise through altered interspecific interactions are largely unexplored.

Here, we report on a study of the direct and indirect effects of anthropogenic sound on predators, their herbivorous prey, and plants. The exact definition of noise pollution is difficult to articulate, but for the purposes of this study, we consider any anthropogenic sound that affects species and their interactions to be noise pollution. We were specifically motivated by the null hypothesis that “Rock and roll ain't noise pollution,” originally posed by the Australian rock band AC/DC (Young, Young, & Johnson, 1980). We tested the direct effects of anthropogenic sounds on growth rates of plants and their herbivorous pests, as well as predation rates of an important biological control species. Although music is not generally considered a threat to ecological systems, popular music was useful as a “proof‐of‐concept” to determine whether anthropogenic sounds can affect interspecific interactions. To generalize our results, we also included a treatment of industrialized/urban sounds to test the hypothesis that anthropogenic sound can affect predator–prey interactions and alter the strength of trophic cascades.

## 2 STUDY SYSTEM

Native to Asia, soybean aphids (*Aphis glycines;* hereafter referred to as “aphids”) were first document in the United States in 2000 (Ragsdale, Landis, Brodeur, Heimpel, & Desneux, 2011; Ragsdale, Voegtlin, & O'Neil, 2004). Their primary summer host is cultivated soybean (*Glycine max*), an important agricultural crop (Ragsdale et al., 2004, 2011). Unfortunately, these aphids have become a major crop pest, altering arthropod communities, and promoting “invasion meltdowns” (Heimpel et al., 2010). The most common predators observed for these pests in North America are the Coccinellidae (Ragsdale et al., 2011). In particular, the introduced multicolored Asian lady beetle (*Harmonia axyridis*; hereafter referred to as “lady beetles”), experienced population facilitation (doubling after the year 2000) in North America after the arrival of soybean aphids (Heimpel et al., 2010). These lady beetles consume proportionally more soybean aphids than any other coccinellid, making them one of the most important sources of biological control of this pest (Costamagna & Landis, 2007).

While the effects of sound pollution on these species have not been specifically investigated, some research has been conducted on related taxa. Collins and Foreman (2001) showed that beans (*Phaseolus* sp.) grew less when exposed to random sound as compared to pure tones, suggesting noise pollution could negatively affect plants directly. Acoustic stimuli across a broad range of frequencies (100–10,000 Hz) have been shown to decrease feeding rates of green peach aphid, *Myzus persicae* (Lee, Kim, Kang, & Jang, 2012). Additionally, some aphids produce sounds for defense or other reasons (Broughton & Harris, 1971; Eastop, 1954; Williams, 1922), and detection of predator vibrations are important for escape behaviors (Francke, Harmon, Harvey, & Ives, 2008), suggesting sound pollution could affect predator–prey interactions in these systems.

## 3 METHODS

We conducted a series of experiments to determine if anthropogenic sound directly affected growth rates of plants and aphids, predation rates by lady beetles, and resulting trophic cascades. All experiments were conducted using laboratory colonies of lady beetles and aphids within plant growth chambers (Percival model E41‐L2, Perry, IA, USA) located in the Department of Biological Sciences at Mississippi State University. All experiments were conducted in a paired design in two different chambers, and then repeated with the chamber treatments switched. For example, during the first trial, Chamber A may be assigned as “control” (i.e., no sound) and Chamber B assigned as “treatment” (i.e., with sound), then in the second trial Chamber A would be assigned as “treatment” and Chamber B as “control.” Although technically pseudoreplication, we treat individual potted plants (experiments A and C) and Petri dishes (experiment B) as experimental units because the growth chambers are controlled environments with identical conditions and that we never saw evidence that the chambers effect plants, aphids, or predators differently. Furthermore, as an additional conservative measure, we included “chamber” as a fixed effect in our analysis to account for between‐chamber differences.

Sound treatments were created using powered computer speakers (Logitech, Romanel‐sur‐Morges, Switzerland, Model Z200) and an iPhone 5 (Apple, Cupertino, CA). Speakers were positioned so that the study organisms were centered between two speakers on a single shelf within the growth chamber. Audio files were assembled into playlists in the software iTunes, and played on “shuffle” (i.e., randomized order of audio tracks) continuously for the duration of each experiment. The software includes a feature to maintain approximately consistent volume among tracks (“Sound Check”). However, we used a sound level meter (Reed Instruments, Wilmington, NC, Model R8050) to confirm that the volumes among sound treatments were similar. The volume of our sound treatments (except for one half‐volume treatment; see section B below for details) was approximately 95–100 dBA; as a comparison, an outboard motor, power lawn mower, jackhammer, and farm tractor are approximately 100 dBA, and a busy urban street or diesel truck is approximately 90 dBA (Brattstrom & Bondello, 1983). Background sound levels in the growth chambers were measured at approximately 70 dBA, indicating our experimental treatments increased sound levels 20 dBA relative to no‐sound controls. While decibel measurements using A‐weighted filters (dBA) are best suited for human perception, more evenly weighted filtering, such as C‐weighted (dBC), are recommended for insect studies (Morley et al., 2014). Thus, also we confirmed our sound treatment levels were similar using a C‐weighted filter (range: 98–106 dBC).

### 3.1 Does sound affect plant biomass or aphid density?

We tested the hypothesis that anthropogenic sound had no effect on (i) plant biomass or (ii) aphid population size in 2‐week experiments. Specifically, we compared control groups (no sound) to treatment groups exposed to the album *Back in Black* by AC/DC continuously for the duration of the experiment. Soybean plants (*n *=* *16) were grown in 1.7 L pots (Belden Plastics, St. Paul, MN) in a common environment until the plants were ~15 cm tall and had multiple true leaves (approximately 2 weeks). We then randomly assigned four plants to each treatment group in a 2 × 2 factorial design crossing sound treatment (control or music) and aphids (absent or present). To maintain independent experimental units, each plant was covered by a clear plastic cylinder (35 cm tall, 10 cm diameter) with an insect mesh lid. Additionally, mesh‐covered openings (5 cm × 7 cm) were cut from the sides of the cylinders to allow unobstructed passage of air and sound. We then initiated the experiment by stocking “aphid present” plants with approximately 50 aphids (range: 47–53). Pots for each sound treatment were placed into a single plastic‐watering tray (Greenhouse Megastore, Danville, IL, Model 1020), with aphid treatments randomly positioned within the tray. We watered each tray approximately 1 L *ad libitum*. Chambers were programed to provide 12 hr of light at 22°C and 12 hr of darkness at 18°C (mean temperature = 20°C).

The experiment was terminated after 14 days. We counted all aphids visible on each plant, then dried plants individually in paper bags at 50°C for 48 hr and weighed them to the nearest milligram. The experiment was repeated using identical methods, except switching growth chamber treatment assignments. We used the R statistical programing environment to analyze all data (R Development Core Team, 2016). To analyze the impact of sound on plant biomass with and without aphids, we used a linear model. Sound treatment, aphid presence, and chamber were each treated as a fixed effect in the model. In all analyses, chamber was not treated as a random effect due to low treatment levels (only two different chambers). The impact of noise on aphid abundance was also analyzed with a linear model, treating treatment and chamber as fixed effects. Inferences from the linear models are based on likelihood ratio tests that compare models with and without a specific fixed effect. Models assumptions were checked using QQ plots and residuals plots.

### 3.2 Does sound affect predation rates?

We tested the null hypothesis that predation rates are unaffected by anthropogenic sound. We tested six different sound treatments: AC/DC's *Back in Black* (full volume and half volume), a compilation of classic Country music, a compilation of popular Rock music, an album by the British folk‐punk band Warblefly, as well as a 17‐track mix of industrial and urban sounds (see Supporting information Appendix S1 for specific track details). All experiments were conducted inside a plant growth chamber set at full light and constant 20°C.

We measured predation rates by placing a single lady beetle larva and a known number of aphids in a Petri dish and counting the number of aphids remaining after a known amount of time. Soybean leaf fragments with a known number of attached aphids (mean 30.7 ± 0.4 SE) were placed into 100 mm diameter Petri dishes with a single 2nd or 3rd instar lady beetle. We then randomly assigned Petri dishes to control or treatment groups, placing them on the top shelf of the appropriate growth chamber. Sample sizes varied among trials due to differences in the availability of lady beetles (range: 6–20). Each sound treatment was conducted twice using identical methods, except switching growth chamber treatment assignments. To analyze the impact of sound on predation rates under various sound treatments, we used a linear model. Treatment, sound, and chamber were each treated as a fixed effect in the model. Unique sounds were then analyzed on an individual basis using treatment and chamber as fixed effects. Inferences and assumptions were evaluated the same as in question A.

### 3.3 Does sound affect trophic cascades?

We tested the hypothesis that anthropogenic sound had no effect on (i) aphid population size or (ii) plant biomass in the presence of lady beetles, using similar methods as that described above (section A). Specifically, we compared control groups (no sound addition) to treatment groups exposed to the album *Back in Black*. Soybean plants were grown in 1.7 L pots in a common environment until the plants were ~15 cm tall and had multiple true leaves (approximately 2 weeks). Each plant was covered by a clear plastic cylinder (described in section A) and inoculated with approximately 50 aphids. After waiting 2 days so that the aphids could establish on their new host plants, we randomly assigned each plant to control or treatment groups. We then initiated the experiment by stocking each plant with a single 2nd instar lady beetle. Chambers were programed to provide 12 hr of light at 22°C and 12 hr of darkness at 18°C (mean temperature = 20°C).

The experiment was terminated after 14 days. We counted all aphids visible on each plant, then dried plants individually in paper bags at 50°C for 48 hr then weighed to the nearest milligram. The experiment was then repeated using identical methods, except switching growth chamber treatment assignments. To analyze the impact of sound on trophic cascades, we used two separate linear models, one for aphid abundance and the second for plant biomass. Treatment and chamber were treated as fixed effects in both models. Inferences and assumptions were evaluated the same as in question A. Aphid abundance was log‐transformed to meet the assumptions of normality.

## 4 RESULTS

### 4.1 No direct effect of anthropogenic sound on plant biomass or aphid density

**Figure 1:**
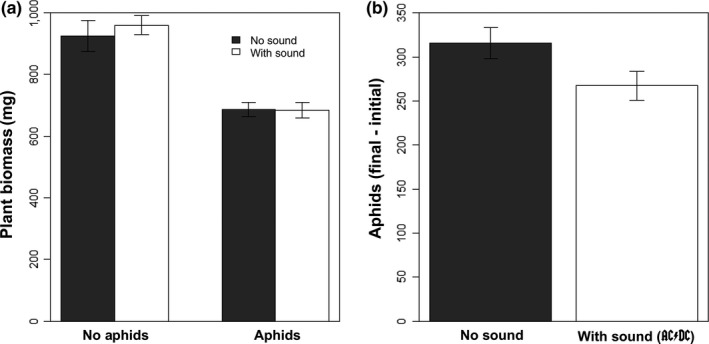
Effects of anthropogenic sound on (a) plant biomass with and without aphids and (b) aphid abundance after 14 days. While aphids significantly reduced plant biomass, sound treatments did not significantly affect plant biomass or aphid abundance in the absence of predators

In both the absence and the presence of aphids, plant biomass (mg) was not impacted by sound treatment (*df* = 1, *F* = 0.227, *p* = 0.637; Figure 1a). Additionally, in the absence of lady beetles, the total number of aphids in each sound treatment was not significantly different (*df* = 1, *F* = 3.863, *p* = 0.0711; Figure 1b).

### 4.2 Reduced predation rates in some anthropogenic sound treatments

**Figure 2:**
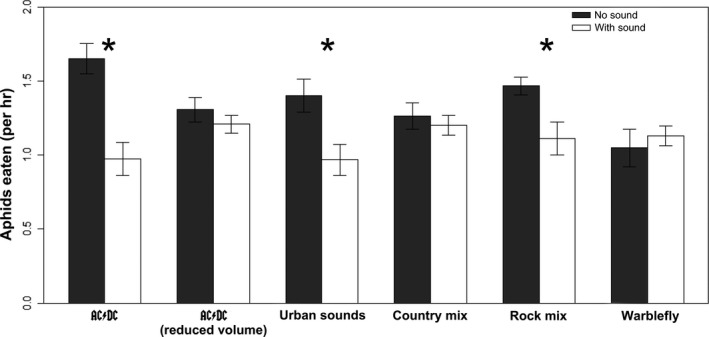
Predation rates of lady beetles on aphids with and without anthropogenic sound. The presence of anthropogenic sound in the form of AC/DC's Back in Black album, a mix of rock music, and a mix of urban sounds reduced predation rates on soybean aphids, whereas other sounds had no effect. * indicate *p *<* *0.05

The number of aphids eaten per hour was not significantly impacted based on chamber (*df* = 1, *F* = 0.210, *p* = 0.647) or sound type (*df* = 5, *F* = 1.340, *p* = 0.247). However, there was a significant difference in aphid consumption between the different treatment types (sound vs. no sound) (*df* = 1, *F* = 25.40, *p* < 0.001; Figure 2). Isolating each different sound type showed that there were significant differences between three of the groups: AC/DC (full volume; *t* = −4.525; *p* < 0.001), City noise (*t* = −2.804, *p* = 0.006), and Rock mix (*t* = −2.837, *p* = 0.006).

### 4.3 Reduced top‐down control in anthropogenic sound treatments

**Figure 3:**
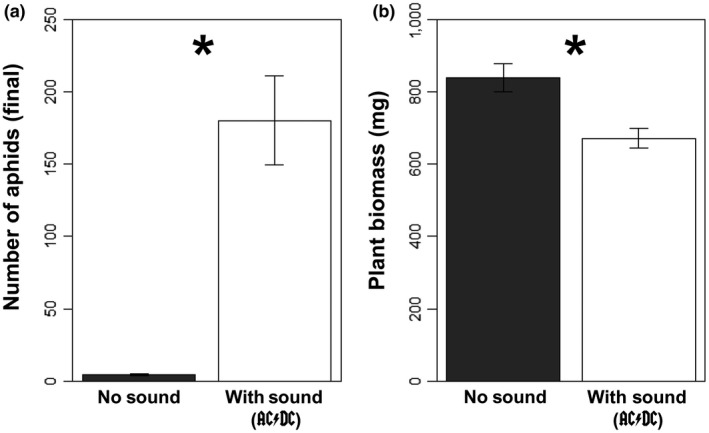
Effect of AC/DC sound treatment on (a) aphid abundance and (b) plant biomass after 14 days. Aphid abundance was significantly higher in the sound treatment. Consequently, plant biomass was higher in no‐sound treatments where aphid abundance was relatively low. * indicate *p *<* *0.05

The presence of lady beetles significantly decreased the number of aphids present in the no‐sound treatments (controls) compared to the treatments with sound (*df* = 1, *F* = 145.63, *p* < 0.001; Figure 3a). In addition, the no‐sound controls had significantly greater plant biomass at the end of the experiment (*df* = 1, *F* = 11.69, *p* = 0.002; Figure 3b).

## 5 DISCUSSION

We found no evidence that sound treatments directly affected plant biomass or aphid density. However, some anthropogenic sounds (e.g., loud rock music and urban sounds) significantly reduced predation rates on aphids in feeding trials. Consequently, when exposed to rock music for 14 consecutive days, relaxed predation rates allowed aphid populations to increase dramatically, suppressing plant biomass relative to music‐free control treatments (Figure 3). Thus, our study found that rock music altered interspecific interactions and generated cascading, indirect effects, and therefore, we reject the AC/DC hypothesis that “rock and roll ain't noise pollution.”

Of course, music is not typically highlighted as a major factor in the increasing pervasiveness of anthropogenic noise. Thus, our study is not meant to precisely replicate the diversity of noise pollution facing natural systems, but instead serve as a “proof‐of‐concept” that anthropogenic sound can generate indirect effects that cascade throughout an ecological community. Most studies of anthropogenic sound focus on the direct individual‐level (e.g., physiological stress) or population‐level (e.g., mate‐finding) effects, with relatively few examples of community‐level indirect effects. Studies have suggested that anthropogenic sounds could alter interactions between predators and prey through mechanisms including antipredator behavior and vigilance (e.g., (Francis et al., 2009; Wale, Simpson, & Radford, 2013; Rabin, Coss, & Owings, 2006)). However, our study uniquely shows that anthropogenic sounds can alter predation rates and indirectly affect prey abundance and plant biomass. Given that species in higher trophic levels are often disproportionately affected by environmental change (Urban, Zarnetske, & Skelly, 2017; Voigt et al., 2003), our results are not surprising and similar patterns of predator‐mediated indirect effects are likely in other systems.

Our results also demonstrate that the effects of noise pollution are not limited to vertebrates, but can affect insects too. Most research on anthropogenic sound has focused on birds and mammals (Radford, Morley, & Jones, 2012), with relatively few studies on insects (Morley et al., 2014). This is problematic given that insects are the most abundant group of animals on the planet (Stork, McBroom, Gely, & Hamilton, 2015; Wilson, 1987), especially in human‐dominated landscapes where noise pollution is most common. Because insects provide a wide range of ecosystem services (Prather et al., 2013), their responses may mediate the net effect of noise pollution on an ecosystem. For example, we found evidence that noise pollution indirectly decreases plant biomass (i.e., soybean yield) because it disrupted biological control of soybean aphids, an important ecosystem service of biodiversity. It is unknown what other insect‐provided ecosystem services are affected by anthropogenic sound, but such investigation would be a laudable next step for future research.

To track the cascading effects of noise pollution across species, our study relied on a relatively simple community of three species, a very controlled laboratory environment, and sound treatment levels that may exceed those found in natural systems. This allowed us to reduce variation and confounding factors that prevent many noise pollution studies from drawing strong conclusions (Radford et al., 2012). However, our study is not without its own limitations. Most importantly, our study was unable to identify the mechanism by which sound treatments altered predation rates. Sound is propagated through the air as waves of pressure, which many organisms receive and detect with ears or analogous structures. However, lady beetles lack any known auditory organ, therefore are likely to perceive sounds differently than mammals, birds, and other “eared” organisms. Thus, although we predict that sound pollution can alter interactions in communities of diverse taxa, the mechanism by which sound induces those effects will likely differ.

Why then did some sound treatments affect lady beetle foraging rates? Several mechanisms could explain our results. First, the effect may not be driven by sound (i.e., vibrations propagated through air) at all, but instead could result from the direct vibration of the speaker housing and transmitted to the experimental units via the substrate (i.e., the growth chamber shelf). Unfortunately, we were unable to quantify substrate vibration, and it is plausible that lady beetles in petri dishes may have been effected through this pathway. However, it seems less plausible that substrate vibration from the vibrating speaker would affect lady beetles foraging on plants. In this case, the direct substrate vibration would have had to travel through the plant pot, through 1.7 L of soil, up the plant stem, and vibrate the leaves where soybean aphids occurred. Although we cannot empirically disprove direct substrate vibration from speakers as a mechanism, it seems unlikely to account for the large and consistent effect of sound in both experiments.

Sound waves traveling from speakers through the air may have affected predators directly or indirectly. Sound waves directly striking lady beetles may have negative effects on them, causing predators to move less, encounter fewer prey, or increase prey handling time. Although *H*. *axyridis* is generally considered a visual predator (Harmon, Losey, & Ives, 1998), it may use vibrational cues to find prey, as has been shown in other beetles (Pfannenstiel, Hunt, & Yeargan, 1995). Alternatively, sound waves directly striking the plants or aphids could have indirect effects on lady beetles. Plants can transmit vibrations between insects (Michelsen, Fink, Gogala, & Traue, 1982), and prey response to vibration could impact predation rates (Clegg & Barlow, 1982). Thus, it is possible that sound waves induced a response or disrupting vibration detection, with consequences for predation rates. Unfortunately, we do not have data to evaluate these potential mechanisms. However, each of these effects could be measured in growth chamber experiments by watching or video recording lady beetles in petri dishes, and we encourage future researchers to explore these mechanisms.

While we found convincing evidence that anthropogenic sound can affect lady beetle foraging rates and indirectly affect aphids and plants, we also show that this does not occur with all anthropogenic sounds. Our results suggest that volume (i.e., magnitude) is important, as illustrated by the large effect of *Back in Black* at a high volume and the absence of an effect from the same music at a lower volume (Figure 2). It remains unclear why some treatments (e.g., Country music and the band Warblefly) failed to have an effect on predation rates when at the same volume as the *Back in Black*, hard rock mix, and urban sound treatments. Our limited technical expertise in evaluating auditory data has precluded us from nuanced characterization of our sound treatments. A quantitative description of sound treatments in terms of frequency content, including spectrum plots, temporal variation, and energy content would facilitate a mechanistic understanding of why some treatments reduced predation rates. Although we are unable to provide such analysis, we encourage future investigators to explicitly consider which aspects of sound are driving these effects to generate a predictive understanding of the effects of anthropogenic sound on communities.

Questioning the effects of sound, particularly rock music, on animal behavior is not new, but has previously focused mostly on human responses. Famously, politicians and activists implicated popular music in malicious human behavior, prompting legislation that requires advisory messages on albums (Gordon, 1989). While the mechanisms (if any) that would lead to changes in human behavior are likely to be quite different for insects, our results show a reduction in antagonistic interactions between lady beetles and their prey. Therefore, while this study shows that rock music has effects on organisms and their interactions, it is important to be clear that there is no evidence of increased aggression or other undesirable behaviors that indicate negative effects of popular music.

In conclusion, we present here the first experimental test of the AC/DC hypothesis since its inception nearly four decades ago (Young et al., 1980). In support of the hypothesis, we found no direct effect of anthropogenic sound on aphids or plants. However, we found clear evidence that some sounds can decrease predation rates of lady beetles thereby indirectly increasing aphid abundance and decreasing plant biomass. Thus, anthropogenic sound initiated a chain of indirect effects that reduced an important ecosystem service of biodiversity, the top‐down control of an agricultural pest by a natural enemy. Our study provides compelling evidence to reject the AC/DC hypothesis, supporting the alternative hypothesis that, in some contexts, rock and roll *is* noise pollution.

## CONFLICT OF INTEREST

None declared.

## DATA ACCESSIBILITY STATEMENT

Data supporting this manuscript will be made available on Dryad following acceptance.

## AUTHOR CONTRIBUTIONS

This study was conceived and designed by BTB. Plants, aphids, and lady beetles were reared by MEH, CJS, AMA, and VPK. Data were collected by BTB, MEH, CJS, AMA, and MAL. CJS conducted data analyses and contributed to writing the manuscript with BTB. VPK and MAL edited the final manuscript, and all authors approve of its submission.
